# Physical inactivity as a risk factor to mortality by ischemic heart disease during economic and political crisis in Brazil

**DOI:** 10.7717/peerj.10192

**Published:** 2020-10-15

**Authors:** Diego Augusto Santos Silva

**Affiliations:** Research Center in Kinanthropometry and Human Performance, Universidade Federal de Santa Catarina, Florianopolis, Santa Catarina, Brazil

**Keywords:** Physical activity, Public health, Kinesiology, Mortality, Heart disease, Cardiology

## Abstract

**Background:**

To investigate the burden of mortality due to ischemic heart disease (IHD) attributable to low levels of physical activity in the Brazilian population (aged ≥ 25 years) before, during and after economic and political crises (2007–2017).

**Methods:**

This study was focused on IHD as a cause of death. The International Statistical Classification of Diseases (10th revision) codes related to IHD have been mapped. The data used for the physical activity estimates of the present study refer to surveys with random sampling carried out in the Brazilian territory that considers all domains of physical activity. The contribution of physical activity for mortality due to IHD was estimated using a comparative risk assessment approach. In addition, we verified the association between mortality due to IHD attributable to low levels of physical activity according to the Socio-demographic Index of the Brazilian states.

**Results:**

For males it was estimated that in 2007 and 2017 there were, respectively, 9,585 and 11,821 deaths due to IHD as a result low physical activity. For females there were 8,689 deaths in 2007 and 10,779 deaths in 2017 due to IHD attributable to low physical activity. From 2007 to 2017, there was 12.0% (for males) and 16.0% (for females) of reduction in age-adjusted mortality rate due to IHD attributable to low physical activity. This reduction was not observed in the Northern and Northeastern regions of Brazil for the male population. Brazilian states with better socioeconomic conditions showed greater reductions in age-adjusted mortality rate due to IHD attributable to low physical activity (male: ρ = −0.74; female: ρ = −0.54)

**Conclusion:**

The fiscal austerity policies implemented and the lower investment in social programs in the period of economic and political crisis highlighted the social inequalities between Brazilian geographic regions for the burden of mortality due to IHD attributable to low levels of physical activity.

## Introduction

Economic and political changes are part of society and bring consequences for population health indicators (Nedel & Bastos, 2020). Policies of economic and social incentives, as was the case in Brazil’s health system in 1988—SUS—which is characterized as universal, free and of quality, directly reflect in the health care of the entire population, regardless of economic and social level of the population (McKee et al., 2012; Paim, 2018; Castro et al., 2019). Since the creation of SUS, health indicators in Brazil have improved (Paim, 2018). On the other hand, economic and political crises bring disastrous results to health, as occurred in the Soviet Union in the late 1980s and the early 1990s, in which the population’s mortality rate increased by about 30% and the life expectancy of people fell from 68 to 58 years of age (Falagas et al., 2009). In addition, Castro et al. (2019) analyzed the 30 years of SUS in Brazil and highlighted that the austerity policies of the last decade are increasing health inequalities in Brazil, which directly impacts the health of the population.

The 2008 economic crisis affected countries in varying magnitudes and durations, particularly as a result of fiscal austerity policies in force (Kentikelenis et al., 2011). In the United Kingdom, the association between suicide and unemployment between 2008 and 2010 was reported (Barr et al., 2012). In Greece, the budget for public hospitals was cut by around 40% and increase in the rates of infectious diseases was observed (Kentikelenis et al., 2011). In Brazil, decrease in the number of preventive cervical screening tests was observed, especially in less socially and economically developed geographic regions (Viana & Silva, 2017).

In addition to the economic crisis of 2008, in the span of a decade (2008–2017), Brazil went through serious structural and political crises (Teixeira & Paim, 2018). One of the most striking aspects of the Brazilian structural and political crisis was the arrest of politicians for corruption and the coup d’état prepared by congressmen that culminated, in 2016, in the impeachment of the President of Brazil (Paim, 2018). Additionally, in this 10-year interval, the unemployment rate in Brazil increased and, especially after 2016, there was reduction in SUS investments and decrease in social policies for people living under conditions of extreme poverty (Paim, 2018).

Ischemic heart disease (IHD) is considered the leading cause of mortality due to cardiovascular diseases worldwide in the adult and elderly population (GBD 2017 Causes of Death Collaborators, 2018). In 2017, it was estimated that approximately 9 million deaths worldwide were due to IHD (GBD 2017 Causes of Death Collaborators, 2018). IHD has multifactorial cause and pharmacological interventions are highly costly to the health system (Schofield et al., 2019). Thus, reductions in mortality rates due to IHD over time may mean greater investments in the prevention and treatment of the disease, which results in advances in health services and in the population’s living conditions (Schofield et al., 2019).

Low levels of physical activity are one of the causes of IHD in the adult and elderly population (Kraus et al., 2019). The dose-response relationship between physical activity and IHD is evident in literature, and a recent review has shown that physical activity protects against this cardiovascular disease and 14% of deaths from this disease could be avoided with the regular practice of physical activity (Kraus et al., 2019). In this sense, investigating the mortality burden due to IHD attributable to low levels of physical activity in a population means obtaining information on how the disease itself and how the causal risk factor have been faced by the country in the prevention and/or treatment of the disease.

This study aims to investigate the mortality burden due to IHD attributable to low levels of physical activity from 2007 (before the economic and political crises) to 2017 (during and after of the crises) in the Brazilian population aged ≥ 25 years from different socially and economically distinct geographic regions.

## Methods

### Study overview

The data in this study correspond to the estimates of the Global Burden of Disease (GBD) study 2017. More information on the history and estimates of the macro-project can be found in the literature (GBD 2017 Causes of Death Collaborators, 2018; GBD 2017 Mortality Collaborators, 2018; GBD 2017 Risk Factor Collaborators, 2018). The protocol of GBD study in Brazil was approved by Ethics Committee on Research with Human Beings of the Federal University of Minas Gerais, and has been conducted in full accordance with ethical principles, including the provisions of the World Medical Association Declaration of Helsinki (Ethical Application Ref: 62803316.7.0000.5149).

Data were collected as previously described in Silva et al. (2019). The physical activity estimates and the analytical method were performed as previously described in Silva et al. (2019).

### Ischemic heart disease estimates

This study was focused on IHD as a cause of death. The International Statistical Classification of Diseases (10th revision, ICD-10) codes related to IHD have been mapped. ICD-10 codes for incidence, morbidity and mortality due to IHD were I20-I25.9 (GBD 2017 Causes of Death Collaborators, 2018). More information about this strategy has been published previously (GBD 2017 Causes of Death Collaborators, 2018).

Input data for mortality estimates due to IHD in Brazil came from the Vital Registry mortality. For all Brazilian states, the quality of data from the vital registries is considered high and close to high-income countries (Ishitani et al., 2017; Queiroz et al., 2017). Previous publications on this project describe the details of this modeling (GBD 2017 Causes of Death Collaborators, 2018; GBD 2017 Mortality Collaborators, 2018; Silva et al., 2019; Naghavi et al., 2010).

### Low physical activity estimates

The information on physical activity in the present study concerns the Brazilian population aged 25 years or older. Only physical activity lasting at least 10 min was considered, across all domains of life (leisure, work, household and transport) (GBD 2017 Risk Factor Collaborators, 2018; Silva et al., 2019). The total metabolic equivalent (MET) minutes per week was calculated considering the frequency, duration and intensity of physical activity according to the recommendations of the compendium of physical activities (Ainsworth et al., 2011).

The data used for the physical activity estimates of the present study refer to surveys with random sampling carried out in the Brazilian territory that considers all domains of physical activity. Information about the surveys used in this research was published previously (GBD 2017 Risk Factor Collaborators, 2018; Silva et al., 2019).

Physical activity level is categorized by total MET-minutes per week using four categories based on rounded values closest to the quartiles of the global distribution of total MET-minutes/week (GBD 2017 Risk Factor Collaborators, 2018). The lower limit (600 MET-min/week) is the recommended minimum amount of physical activity to get any health benefit (World Health Organization, 2010). More details on the modeling in each of the surveys used in this study can be found in detail in the literature (GBD 2017 Risk Factor Collaborators, 2018; Silva et al., 2019).

### Analytical methods

For this study we used a theoretical minimum-risk exposure level for physical activity of 3000-4500 MET-min per week, which was calculated as the exposure at which minimal deaths across outcomes occurred (GBD 2017 Risk Factor Collaborators, 2018; Kyu et al., 2016). For this, the simulation model by CODEm was used to estimate indicators by age, sex, country, state, year, and cause. Previous studies provide more explanations about the proposed models (GBD 2017 Risk Factor Collaborators, 2018; Silva et al., 2019).

Incident IHD cases, summary exposure variable (SEV) to low physical activity (Silva et al., 2019), absolute number of deaths, mortality rate (per 100,000 inhabitants—crude and age-standardized), and population-attributable fraction (PAF) (GBD 2017 Risk Factor Collaborators, 2018) of death due to IHD related to low physical activity were used as metrics. In the tables/figures of this article, for better visualization, the information was presented for the years 2007 and 2017, however, for the calculation of changes over time, the entire historical series from 2007 to 2017 (i.e., 2007, 2008, 2009, 2010, 2011, 2012, 2013, 2014, 2015, 2016, and 2017) was considered. More details of historical series are available in the literature (GBD 2017 Risk Factor Collaborators, 2018).

We also analyzed the mortality IHD attributed to low physical activity according to Socio-demographic Index (SDI) of the Brazilian states. Information on how to calculate the SDI can be found in the literature (GBD 2017 Mortality Collaborators, 2018).

We report all point estimates with 95% uncertainty intervals (UIs). To ensure that UIs capture uncertainty from all relevant sources we propagate uncertainty through the estimation chain using posterior simulation using 1,000 draws, from which we derive the lower and upper bounds of the UI based on the 2.5th and 97.5th percentiles. Where reported, estimates of percentage change were computed on the basis of the point estimates for the time points being compared (GBD 2017 Mortality Collaborators, 2018; GBD 2017 Risk Factor Collaborators, 2018). Additionally, we used Spearman’s correlation coefficient to verify the association between the age-standardized mortality rate and the SDI of Brazilian states.

The results of the present study were presented by year, sex, state, and Brazilian geographic regions. Brazil is divided into five geographic regions that have the following Human Development Index (HDIs): higher HDI (Mid-Western = 0.753, Southeast = 0.753, Southern = 0.756), lower HDI (Northern = 0.683, Northeastern = 0.659). This form of classification is used in studies of social inequalities in Brazil (Szwarcwald et al., 2016).

## Results

From 2007 to 2017, there was 15% reduction in the incidence of IHD in males in Brazil. The Northern region of Brazil did not show reduction in the incidence of IHD in males from 2007 to 2017 (Table S1). From 2007 to 2017, there was 18% reduction in the incidence of IHD in females in Brazil. In all Brazilian geographic regions, there was reduction in the incidence of IHD in females from 2007 to 2017 (Table S2).

The Brazilian male population presented risk of exposure to low levels of physical activity (age-standardized SEV) of 57.9% in 2007 and 58.1% in 2017 (Table S3). The Brazilian female population presented risk of exposure to low levels of physical activity (age-standardized SEV) of 59.1% in 2007 and 59.2% in 2017 (Table S4).

For males (Table 1), it was estimated that in 2007 and 2017, there were, respectively, 9,585 (95% UI [4,455–15,743]) and 11,821 (95% UI [5,541–19,458]) deaths due to IHD as a result low levels of physical activity. These values represented for males an age-standardized mortality rate per 100,000 inhabitants of 14.2 (95% UI [6.6–23.3]) in 2007 and 12.4 (95% UI [5.8–20.5]) in 2017. From 2007 to 2017, there was 12.0% reduction in mortality rate due to IHD attributable to low levels of physical activity in the Brazilian male population. However, the Northern and Northeastern regions of Brazil did not show reduction from 2007 to 2017.

**Table 1:** Number and age-standardized mortality rate (per 100,000 inhabitants) for ischemic heart disease due to low levels of physical activity in males from Brazil, and Brazilian states in 2007 and 2017 in ages ≥ 25 years.

	2007			2017			2007			2017			Change		
	Number	95%	U.I.	Number	95%	U.I.	Rate*	95%	U.I.	Rate*	95%	U.I.	% rate*	95%	U.I.
Brazil	9,585	4,495	15,743	11,821	5,541	19,548	14.2	6.6	23.3	12.4	5.8	20.5	−12.0	−16.0	−10.0
Northen	433	203	710	652	304	1,073	11.7	5.4	19.2	10.8	5.0	17.9	−6.7	−16.1	3.1
Acre	20	9	32	26	12	44	10.9	5.0	17.8	9.9	4.5	16.6	−9.0	−17.0	−1.0
Amapá	11	5	18	20	10	33	10.2	4.8	16.8	10.5	4.9	17.2	3.0	−5.0	10.0
Amazonas	70	33	115	104	48	171	9.3	4.4	15.3	9.0	4.2	14.8	−3.0	−11.0	4.0
Pará	211	99	347	334	157	544	10.9	5.1	17.9	11.4	5.4	18.7	5.0	−2.0	12.0
Rondônia	60	28	97	85	39	142	13.9	6.5	22.4	13.1	6.0	21.6	−6.0	−21.0	13.0
Roraima	10	5	17	16	7	27	15.2	6.9	25.2	11.8	5.5	19.9	−22.0	−34.0	−10.0
Tocantins	51	24	84	67	31	112	11.6	5.4	19.0	9.9	4.5	16.5	−15.0	−23.0	−6.0
Northeastern	2,399	1,118	3,915	3,123	1,457	5,146	12.4	5.8	20.3	12.3	5.8	20.3	−1.0	−9.1	7.6
Alagoas	123	59	204	161	75	267	12.5	6.0	20.7	12.2	5.7	20.3	−2.0	−9.0	5.0
Bahia	607	280	987	786	369	1,280	11.6	5.4	18.9	11.5	5.4	18.6	−1.0	−10.0	8.0
Ceará	352	163	577	431	195	720	11.1	5.2	18.2	10.0	4.6	16.7	−9.0	−16.0	−1.0
Maranhão	252	118	418	359	170	596	12.2	5.7	20.2	12.8	6.1	21.3	5.0	−3.0	15.0
Paraíba	232	108	374	269	124	443	14.3	6.7	23.1	13.5	6.2	22.3	−6.0	−16.0	5.0
Pernambuco	475	223	775	632	297	1,039	15.3	7.1	24.9	15.6	7.4	25.7	2.0	−6.0	10.0
Piauí	148	69	239	183	85	299	12.7	5.9	20.5	11.4	5.3	18.6	−10.0	−16.0	−4.0
Rio Grande do Norte	142	66	230	206	97	344	11.6	5.4	18.8	12.7	6.0	21.2	10.0	1.0	20.0
Sergipe	68	32	111	96	45	158	10.7	5.0	17.5	10.9	5.1	17.9	2.0	−7.0	10.0
Mid-Western	560	264	919	732	347	1,210	14.4	6.7	23.6	11.9	5.6	19.5	−17.8	−23.8	−11.8
Distrito Federal	73	34	118	95	45	156	16.2	7.4	26.2	12.0	5.5	19.6	−26.0	−32.0	−20.0
Goiás	241	115	397	315	150	522	12.3	5.8	20.2	10.7	5.1	17.7	−13.0	−19.0	−7.0
Mato Grosso	114	53	187	150	71	248	12.8	5.9	20.9	10.7	5.0	17.5	−17.0	−23.0	−11.0
Mato Grosso do Sul	132	62	217	172	81	284	16.4	7.7	27.0	14.0	6.6	23.0	−15.0	−21.0	−9.0
Southeast	4,686	2,194	7,682	5,550	2,610	9,107	15.7	7.4	25.7	12.7	6.0	20.8	−19.0	−24.5	−14.3
Espírito Santo	180	85	292	195	93	316	14.8	6.9	24.1	10.9	5.2	17.7	−26.0	−31.0	−22.0
Minas Gerais	919	430	1,506	1,082	505	1,782	11.7	5.5	19.2	9.7	4.6	16.0	−17.0	−24.0	−12.0
Rio de Janeiro	1,237	586	2,007	1,462	693	2,379	20.0	9.4	32.5	16.9	8.0	27.5	−15.0	−20.0	−10.0
São Paulo	2,350	1,093	3,877	2,811	1,319	4,630	16.3	7.6	26.9	13.4	6.2	22.0	−18.0	−23.0	−13.0
Southern	1,510	714	2,479	1,764	834	2,911	14.8	6.9	24.3	12.0	5.7	19.9	−18.7	−23.7	−14.0
Paraná	566	270	928	662	314	1,084	14.9	7.0	24.4	12.4	5.9	20.3	−17.0	−22.0	−12.0
Rio Grande do Sul	650	306	1,065	740	349	1,227	14.9	7.0	24.5	12.1	5.7	20.1	−19.0	−24.0	−14.0
Santa Catarina	294	138	486	362	171	600	14.6	6.8	24.0	11.6	5.5	19.4	−20.0	−25.0	−16.0

**Note:**

* Age-standardized rate; U.I.: uncertainty interval.

For females (Table 2), there were 8,689 (95% UI [3,962–14,103]) deaths in 2007 and 10,779 deaths in 2017 (95% UI [4,895–17,472]) due to IHD attributable to low levels of physical activity. These values represented for females an age-standardized mortality rate of 10.3 (95% UI [4.7–16.7]) in 2007 and 8.6 (95% UI [3.9–14.0]) in 2017 per 100,000 inhabitants. From 2007 to 2017, there was 16.0% reduction in mortality rate due to IHD attributable to low levels of physical activity in the Brazilian female population. In all Brazilian geographic regions, there was reduction in the mortality rate due to IHD attributable to the low levels of physical activity in the female population.

**Table 2:** Number and age-standardized mortality rate (per 100,000 inhabitants) for ischemic heart disease due to low levels of physical activity in female from Brazil, and Brazilian states in 2007 and 2017 in ages ≥ 25 years.

	2007			2017			2007			2017			Change		
	Number	95%	U.I.	Number	95%	U.I.	Rate*	95%	U.I.	Rate*	95%	U.I.	% rate*	95%	U.I.
Brazil	8,689	3,962	14,103	10,779	4,895	17,472	10.3	4.7	16.7	8.6	3.9	14.0	−16.0	−19.0	−14.0
Northen	322	146	527	446	199	729	9.4	4.2	15.3	7.6	3.4	13.0	−16.7	−24.9	−7.4
Acre	14	6	22	19	8	30	8.3	3.7	13.5	6.9	3.1	11.2	−17.0	−24.0	−9.0
Amapá	8	4	13	14	6	24	7.0	3.2	11.3	6.6	2.9	11.0	−6.0	−16.0	6.0
Amazonas	54	24	88	78	35	127	6.9	3.1	11.2	6.3	2.9	10.4	−8.0	−15.0	0.0
Pará	162	74	266	213	96	347	7.8	3.6	12.8	6.7	3.0	10.9	−14.0	−21.0	−8.0
Rondônia	38	17	63	59	26	100	12.1	5.4	19.8	9.8	4.3	16.4	−19.0	−30.0	−5.0
Roraima	6	3	10	10	4	16	13.7	6.0	22.4	9.0	3.9	14.8	−34.0	−43.0	−25.0
Tocantins	40	18	65	53	24	85	10.1	4.6	16.4	8.2	3.7	16.4	−19.0	−25.0	−11.0
Northeastern	2,230	1,007	3,620	2,795	1,262	4,575	9.3	4.2	15.0	8.4	3.8	13.8	−9.2	−17.0	−0.9
Alagoas	122	56	197	168	76	273	10.2	4.6	16.4	10.0	4.6	16.3	−1.0	−9.0	7.0
Bahia	565	252	913	634	287	1,032	8.7	3.9	14.1	7.0	3.2	11.3	−21.0	−28.0	−11.0
Ceará	345	154	558	453	200	746	8.5	3.8	13.8	7.9	3.5	13.0	−8.0	−16.0	0.0
Maranhão	172	80	285	207	97	343	7.4	3.4	12.2	6.2	2.9	10.2	−16.0	−24.0	−7.0
Paraíba	228	103	369	283	126	471	10.9	5.0	17.7	10.2	4.6	17.0	−6.0	−17.0	6.0
Pernambuco	468	213	763	620	281	1,007	11.4	5.2	18.5	11.0	5.0	17.9	−3.0	−10.0	4.0
Piauí	126	57	203	153	70	249	8.9	4.0	14.3	7.5	3.4	12.2	−15.0	−21.0	−8.0
Rio Grande do Norte	131	59	213	185	83	303	8.3	3.8	13.6	8.4	3.7	13.7	1.0	−8.0	9.0
Sergipe	73	33	119	92	42	151	9.1	4.1	14.8	7.8	3.5	12.8	−14.0	−20.0	−8.0
Mid-Western	444	201	719	570	259	921	11.1	5.0	18.1	8.2	3.7	13.3	−26.0	−31.8	−20.5
Distrito Federal	59	27	95	72	32	118	11.2	5.1	18.1	7.3	3.3	11.9	−35.0	−41.0	−29.0
Goiás	208	94	336	266	121	429	10.8	4.8	17.5	8.3	3.8	13.4	−23.0	−28.0	−18.0
Mato Grosso	79	35	127	101	47	161	10.4	4.6	16.8	7.7	3.5	12.3	−25.0	−31.0	−19.0
Mato Grosso do Sul	98	45	161	131	59	213	12.1	5.5	19.8	9.6	4.3	15.5	−21.0	−27.0	−16.0
Southeast	4,232	1,917	6,893	5,215	2,371	8,447	10.9	4.9	17.6	8.7	3.9	14.0	−20.8	−25.8	−15.3
Espírito Santo	154	70	250	167	75	272	10.8	4.9	17.5	7.4	3.3	12.0	−32.0	−37.0	−27.0
Minas Gerais	842	380	1,377	1,017	460	1,652	8.7	3.9	14.3	7.2	3.2	11.6	−18.0	−23.0	−12.0
Rio de Janeiro	1,104	501	1,775	1,372	629	2,218	12.4	5.6	19.9	10.7	4.9	17.3	−13.0	−18.0	−7.0
São Paulo	2,132	966	3,491	2,659	1,207	4,305	11.5	5.2	18.8	9.3	4.2	15.0	−20.0	−25.0	−15.0
Southern	1,461	662	2,368	1,755	788	2,847	11.4	5.1	18.4	9.1	4.1	14.7	−20.0	−24.7	−14.7
Paraná	504	229	824	621	282	1,006	12.0	5.4	19.5	9.5	4.3	15.3	−21.0	−25.0	−15.0
Rio Grande do Sul	697	317	1,123	791	352	1,288	11.5	5.2	18.5	8.9	4.0	14.5	−22.0	−27.0	−18.0
Santa Catarina	260	116	421	343	154	553	10.6	4.7	17.1	8.8	4.0	14.2	−17.0	−22.0	−11.0

**Note:**

* Age-standardized rate; U.I.: uncertainty interval.

**Figure 1:**
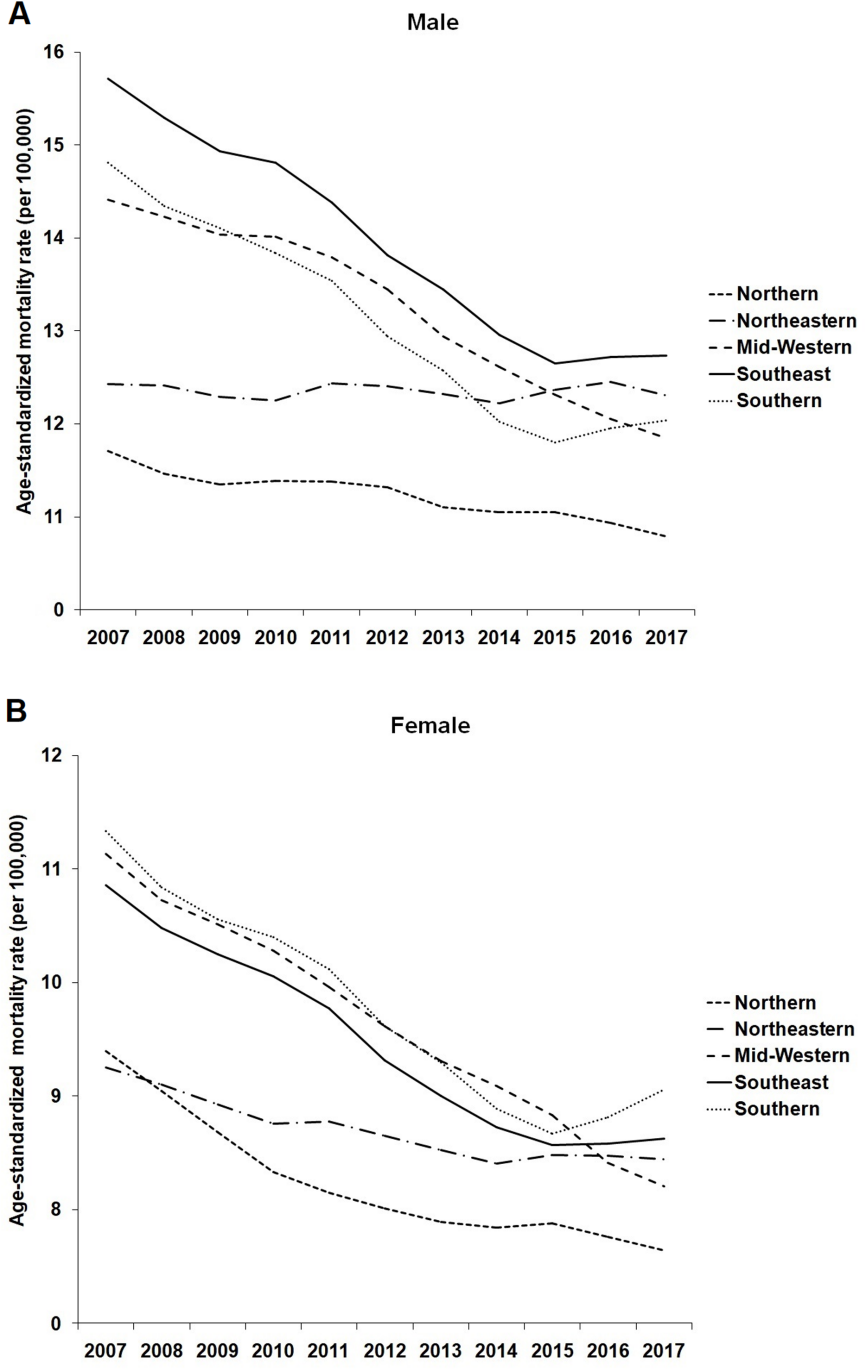
Age-standardized mortality rate (per 100,000 inhabitants) for ischemic heart disease due to low levels of physical activity in male (A) and female (B) from Brazil according to geographic region (2007–2017).

**Figure 2:**
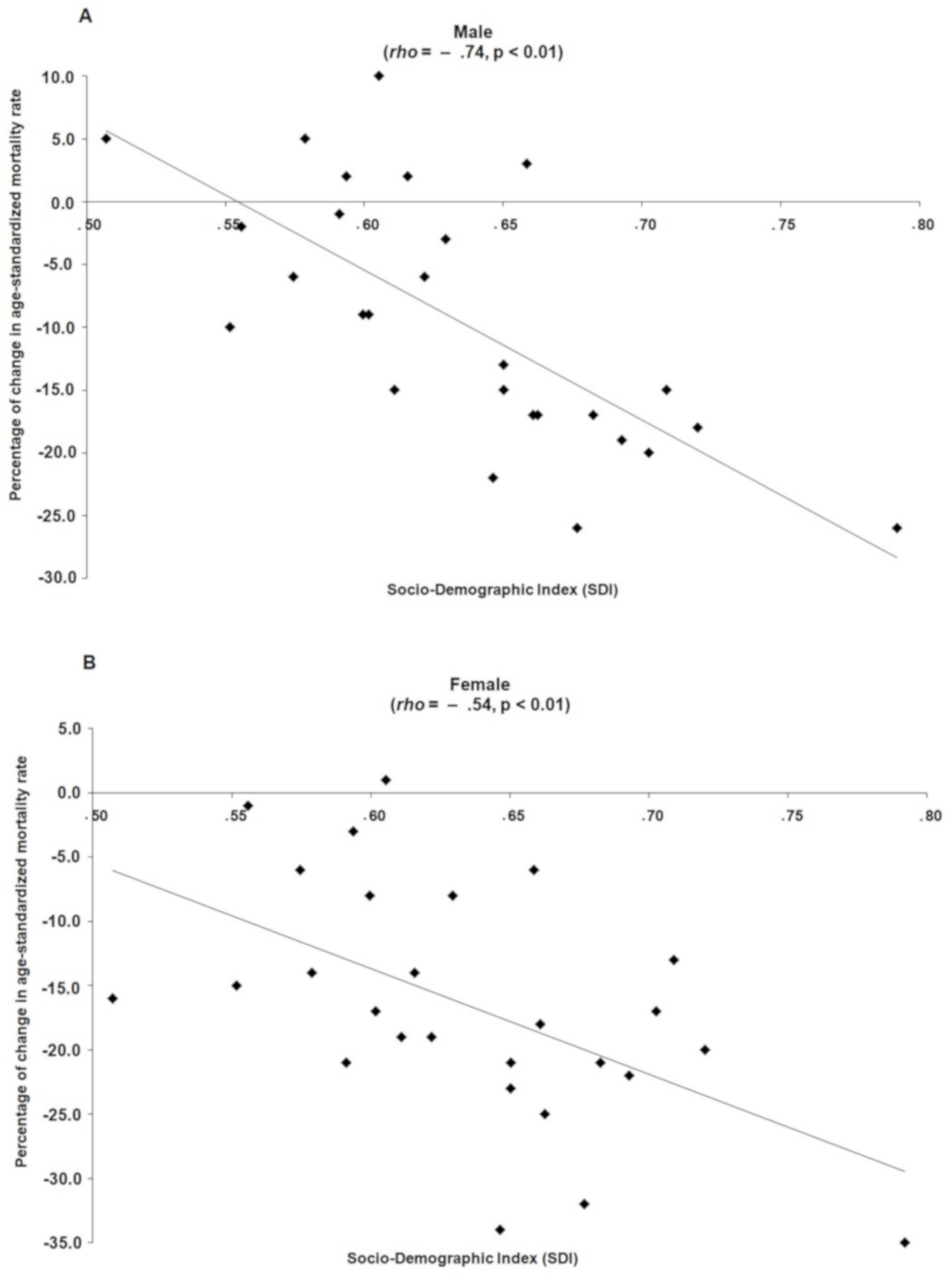
Relation between Socio-demographic index of the Brazilian states with the percentage of change from 2007 to 2017 in the age-standardized mortality rate for ischemic heart disease due to low levels of physical activity in male (A) and female (B).

In 2007, the highest age-standardized mortality rates due to IHD attributable to low levels of physical activity were estimated, respectively, for the Southeastern, Southern and Midwestern regions for males (Fig. 1A) and, in the Southern, Midwestern and Southeastern regions for females (Fig. 1B). Over the 11 years of this study (2007–2017), in both sexes, this scenario changed and the greatest reductions in age-standardized mortality rates due to IHD attributable to low levels of physical activity were observed in the Midwestern, Southern and Southeastern regions of Brazil and the lowest reductions in the Northern and Northeastern regions (Figs. 1A and 1B). In addition, Brazilian states with better socioeconomic conditions showed greater reductions in age-standardized mortality rates due to IHD attributable to low physical activity (Male: ρ = −0.74; Female: ρ = −0.54) from 2007 to 2017 (Fig. 2).

In 2007 and 2017, in the male population aged 25–49 years, 50–69 years and ≥70 years, approximately 10%, 12% and 13%, respectively of deaths from IHD could be avoided with regular practice of physical activity. In 2007 and 2017, in the female population aged 25-49 years, 50-69 years and ≥70 years, approximately 12%, 13% and 14%, respectively of deaths from IHD could be avoided with regular practice of physical activity. These results were similar in all geographic regions of Brazil (Table 3).

**Table 3:** Population-attributable fraction of the deaths due to ischemic heart disease attributed to low physical activity in the Brazilian population according to age in 2007 and 2017.

	Male				Female			
	PAF (95% U.I.)				PAF (95% U.I.)			
	All ages*	25–49 years	50–69 years	≥70 years	All ages*	25–49 years	50–69 years	≥70 years
2007								
Brazil	12.1 [5.7–20.0]	9.9 [4.6–16.6]	11.5 [5.4–19.1]	12.8 [6.0–21.1]	13.8 [6.2–22.4]	11.9 [5.6–19.6]	13.3 [6.2–21.6]	14.1 [6.4–22.8]
Northen	12.0 [5.6–19.8]	9.5 [4.4–15.7]	11.3 [5.3–25.1]	12.6 [5.9–20.8]	13.7 [6.2–22.3]	11.7 [5.4–19.1]	13.3 [6.1–21.6]	14.0 [6.3–19.9]
Northeastern	11.8 [5.5–19.4]	9.4 [4.3–15.6]	11.2 [5.2–18.6]	12.5 [5.9–20.6]	13.4 [6.1–21.7]	11.4 [5.3–18.7]	12.9 [6.0–21.1]	13.8 [6.2–22.3]
Mid-Western	12.2 [5.7–20.1]	9.9 [4.6–16.4]	11.4 [5.4–18.9]	12.8 [6.0–21.1]	13.9 [6.3–22.6]	12.1 [5.6–19.8]	13.5 [6.2–21.8]	14.2 [6.4–23.0]
Southeast	12.3 [5.8–20.2]	10.2 [4.8–17.0]	11.6 [5.5–19.2]	12.9 [6.0–21.2]	13.8 [6.2–22.4]	12.1 [5.6–19.7]	13.4 [6.1–21.7]	14.2 [6.4–22.9]
Southern	12.1 (5.6–19.8)	9.8 (4.6–16.3)	11.3 (5.3–18.8)	12.7 (5.9–20.8)	13.9 (6.3–22.5)	12.1 (5.6–19.7)	13.3 (6.1–21.6)	14.1 (6.4–22.8)
2017								
Brazil	12.2 [5.7–20.1]	9.9 [4.6–16.4]	11.6 [5.5–19.3]	12.9 [6.0–21.2]	13.8 [6.3–22.4]	11.9 [5.6–19.5]	13.4 [6.2–21.8]	14.2 [6.4 -22.9]
Northen	12.1 [5.7–20.0]	9.5 [4.4–15.7]	11.4 [5.4–18.9]	12.7 [5.9–20.9]	13.7 [6.2–22.3]	11.7 [5.4–19.0]	13.3 [6.1–21.7]	14.0 [6.3–22.7]
Northeastern	11.9 [5.6–19.7]	9.4 [4.3–15.6]	11.3 [5.3–18.7]	12.6 [5.9–20.7]	13.4 [6.1–21.8]	11.5 [5.3–18.8]	13.0 [6.0–21.2]	13.8 [6.2–22.4]
Mid-Western	12.2 [5.7–20.2]	9.9 [4.6–16.4]	11.5 [5.5–19.1]	13.0 [6.0–21.2]	14.0 [6.3–22.7]	12.1 [5.6–19.8]	13.5 [6.2–22.0]	14.3 [6.4–23.1]
Southeast	12.4 [5.8–20.4]	10.1 [4.7–16.8]	11.8 [5.5–19.5]	13.1 [6.0–21.3]	13.8 [6.2–22.5]	12.1 [5.6–19.7]	13.5 [6.1–21.8]	14.2 [6.4–22.9]
Southern	12.1 [5.7–19.9]	9.8 [4.5–16.2]	11.4 [5.3–18.9]	12.7 [5.9–20.8]	13.9 [6.3–22.5]	12.0 [5.6–19.7]	13.4 [6.2–21.7]	14.2 [6.5–22.9]

**Note:**

* Age-standardized; U.I.: uncertainty interval; PAF: Population-attributable fraction.

## Discussion

Data obtained in the present study revealed that there was no increase in mortality rates due to IHD attributable to low levels of physical activity related to economic and political crisis. A justification for this finding may be the greater control that the Brazilian health system has exercised in recent years over risk factors for cardiovascular diseases, such as hypertension and smoking, in addition to better treatment conditions for acute cardiovascular events (Brant et al., 2017). In addition, even with economic and political crises, SUS is a reference in the Primary Care network and, therefore, in the primary and secondary prevention of cardiovascular diseases (Brant et al., 2017). Castro et al. (2019) proposed an analysis of public investments in SUS in different scenarios, among them a scenario that is more present today, which is the lowest investment of the Brazilian government in SUS. Castro et al. (2019) predictions are that if the investment of the Brazilian government remains the same as that employed today, the cardiovascular disease mortality will stop decreasing, which will lead to a setback for the country.

During and after the economic and political crisis, the most socially and economically favored Brazilian states reduced mortality rates due to IHD attributable to low levels of physical activity and the least socially and economically favored regions and states in Brazil stagnated in this process (males) or reduced to a lesser extent (females) compared to the most socially and economically favored regions and states. These results demonstrate social inequalities and the impact of fiscal austerity policies on Brazilian regions and states, reflecting the inequality in the fight against IHD and low levels of physical activity in the population. Situations of fiscal austerity in times of economic and political crisis reflect on deeper inequalities and worsening of health indexes, especially actions that burden the population with the increase in taxes and reduction of social rights (Santos & Vieira, 2018). An important issue in Brazil is income inequality, which is strongly determined by the tax system, which is one of the most regressive in the world (Santos & Vieira, 2018). A study carried out with data from 2006 to 2012 identified that the richest strata of Brazilian society explained the high social inequality in the country due to the tax system (Medeiros & Souza, 2016). In Brazil, the richest strata are concentrated in the Southeastern, Southern and Midwestern geographic regions (Szwarcwald et al., 2016), precisely the regions that, in the present study, presented greater decrease in mortality rates due to IHD attributable to low levels of physical activity from 2007 to 2017.

In economic terms, a series of movements affected Brazil with the 2008 crisis, such as the dollar variation, lack of money available for bank loans, inflation, low and high interest rates and retraction of the economy in 2009. The Brazilian economic crisis, aggravated from 2014 onwards with more evident structural and political crises, had as immediate effects the drop in tax revenues in all spheres of government and increase in unemployment (Santos & Vieira, 2018). The approval in 2016 of a Constitutional Amendment (EC No. 95) froze public spending in the social area, including health, for the next 20 years. This directly reflected in the supply and quality of the health system in Brazil and prioritized private health insurances and hospitals (Teixeira & Paim, 2018). In practice, the fiscal adjustment implemented in Brazil did not have as main objective to control the momentary imbalance in public accounts, but rather to force the reduction of the State’s participation in the supply of goods and services to the population through the reduction of spending on public policies (Teixeira & Paim, 2018). This situation is lethal for less socially and economically developed regions, for this reason, one of the explanations for the results of stagnation or decrease in lower magnitude of mortality rates due to IHD attributable to the low levels of physical activity in the Northern and Northeastern regions of the country in comparison with the most socially and economically developed regions were the fiscal austerity policies implemented in Brazil with the economic and political crisis.

In periods of economic and political crisis, the population has increased levels of stress and depression due to uncertainties of the future that include changes in family life and work (Falagas et al., 2009). This psychological status of uncertainties, marked stress and depression bring emotional responses associated with mortality due to cardiovascular events (Falagas et al., 2009). Physiologically, increased levels of circulating catecholamines in response to stressful episodes can precipitate acute cardiovascular events (Gidron et al., 2002; Smoller et al., 2007). In addition, changes in mood-related neuroendocrine and immunological responses and mental changes due to stress and depression contribute to the instability of atherosclerotic plaque and thrombogenesis (Gidron et al., 2002; Kop & Gottdiener, 2005). In this sense, cardiovascular events in times of economic and political crises affect populations of less favored social and economic locations in greater magnitude, or as was the case in the present study, the incidence of IHD and mortality rate due to IHD attributable to low levels of physical activity remained stable or decreased to a lesser extent from 2007 to 2017 in the Northern and Northeastern regions of Brazil compared to other regions.

The biological mechanisms by which regular physical activity reduces the risk of cardiovascular diseases can be summarized as follows: physical activity reduces blood pressure, improves the blood lipid profile, and decreases systemic inflammation and decreases damage and atherosclerosis of cardiac, cerebral, and peripheral blood vessels. Physical activity also improves endothelial function and has antithrombotic effect, which further reduces the risk of adverse cardiac and cerebrovascular events (Bouchard, Blair & Haskell, 2012). The present study demonstrated that from 2007 to 2017, there were no changes in the prevalence of the Brazilian population exposed to the risk of low levels of physical activity. A possible explanation for the stagnation during a 10-year period of the Brazilian population in relation to physical activity is that the economic and political crisis in Brazil negatively influenced the scope of public policies to promote physical activity in that period. In a scenario of economic austerity, healthy lifestyle behaviors are difficult to achieve (Cortès-Franch & González López-Valcárcel, 2014).

The present study found that regardless of geographic region, approximately 12% of deaths due to IHD could be avoided in 2007 and in 2017 if the Brazilian population had practiced physical activity on a regular basis. These data reveal that the practice of physical activity is important for the prevention of mortality due to IHD in populations from different economic, social and age groups, regardless of political and economic crises. Physical activity should be on the health promotion policy agenda of Brazil and greater investments should be prioritized in this field. However, with the structural and political crisis that affected Brazil in the period studied in this article, investments in health have been frozen for 20 years (Santos & Vieira, 2018), which may reflect less investment in policies aimed at the promotion of physical activity in the next decade. In other words, lack of investment or stagnation in investments in public policies to promote physical activity ceases to make the population more physically active and fails to prevent 12% of deaths due to IHD in the Brazilian population.

The present study has the limitation of having considered only one type of cardiovascular disease in the mortality estimation, considering that regular physical activity can be beneficial for other types of cardiovascular diseases (Kraus et al., 2019). Another limitation was the failure to analyze the economic austerity measures taken by each Brazilian state in the specific period of economic crisis, as each Brazilian state could, for example, have different taxation measures in relation to the federal government. Another limitation of this research was the calculation of physical activity only by surveys that used self-reported measures, whose estimates have higher risk of bias compared to objective measures (Silva et al., 2018). Other limitations of estimation models are evidenced in literature (GBD 2017 Risk Factor Collaborators, 2018).

## Conclusion

It could be concluded that less socially and economically favored Brazilian states and geographic regions had reductions in mortality rates due to IHD attributable to low levels of physical activity in lesser magnitude (females) or were stagnant (males) in the period under study in comparison to the most socially and economically developed geographic regions. Fiscal austerity policies and lower investments in social programs in the period of economic and political crisis highlighted social inequities in Brazil and reflected in mortality rates due to IHD attributed to low levels of physical activity in the population aged ≥25 years.

## Supplemental Information

10.7717/peerj.10192/supp-1 Supplemental Information 1 Incidence of ischemic heart disease in Brazilian male population in the years 2007 and 2017. *Age-standardized rate; U.I.: uncertainty interval.

10.7717/peerj.10192/supp-2 Supplemental Information 2 Incidence of ischemic heart disease in Brazilian female population in the years 2007 and 2017. Incidence of ischemic heart disease in Brazilian female population in the years 2007 and 2017

10.7717/peerj.10192/supp-3 Supplemental Information 3 Age-standardized summary exposure value to physical inactivity in the Brazilian male population in 1990 and 2017 in ages ≥ 25 years. *Age-standardized rate; U.I.: uncertainty interval; SEV: summary exposure value.

10.7717/peerj.10192/supp-4 Supplemental Information 4 Age-standardized summary exposure value to physical inactivity in the Brazilian female population in 1990 and 2017 in ages ≥ 25 years. *Age-standardized rate; U.I.: uncertainty interval; SEV: summary exposure value.

10.7717/peerj.10192/supp-5 Supplemental Information 5 Dataset. The number and standardized mortality rate and information from all states and geographic regions of the country.

## Additional Information and Declarations

### Data Availability

The following information was supplied regarding data availability: The raw measurements are available in the Supplemental File.
