# Supplementary material for: Physical inactivity as a risk factor to mortality by ischemic heart disease during economic and political crisis in Brazil
Source: PeerJ. 2020 Oct 15;8:e10192. doi: 10.7717/peerj.10192 (PMC7568855; doi:10.7717/peerj.10192)
Supplement: Supplemental Information 1 — *Age-standardized rate; U.I.: uncertainty interval. [file peerj-08-10192-s001.pdf]

Supplemental Table S1. Incidence of ischemic heart disease in Brazilian male population in the years 2007 and 2017.

	2007			2017			2007			2017			Change		
	**Number**	**95% U.I.**		**Number**	**95% U.I.**		**Rate***	**95% U.I.**		**Rate***	**95% U.I.**		**% rate***	**95% U.I.**	
**Brazil**	87,893	78,323	98,486	104,655	91,455	119,062	123.3	110.6	137.3	104.2	91.2	118.6	-15.0	-18.0	-13.0
**Northen**	4,274	3,689	4,887	6,144	5,281	7,077	96.4	83.3	111.1	91.4	78.2	106.0	-5.4	-10.4	0.0
Acre	194	167	222	269	230	311	99.7	85.6	115.0	93.0	79.4	108.9	-7.0	-12.0	-1.0
Amapá	125	107	145	210	179	243	91.9	79.1	106.0	89.5	76.7	104.0	-3.0	-8.0	2.0
Amazonas	791	681	906	1,159	985	1,338	92.1	79.6	106.3	87.9	74.6	102.0	-5.0	-10.0	1.0
Pará	2,115	1,832	2,408	3,013	2,606	3,462	100.0	87.1	114.7	94.8	81.8	109.9	-5.0	-10.0	0.0
Rondônia	486	418	559	678	581	784	99.2	85.9	114.0	92.5	79.2	106.8	-7.0	-12.0	-2.0
Roraima	93	79	108	159	136	184	93.3	80.2	108.1	88.1	75.6	102.0	-6.0	-11.0	0.0
Tocantins	470	405	539	656	564	755	98.9	85.3	113.4	93.7	80.4	108.6	-5.0	-10.0	0.0
**Northeastern**	22,654	18,905	25,677	26,683	24,309	32,191	109.4	95.5	124.6	101.2	87.4	116.3	-7.6	-12.4	-2.2
Alagoas	1,084	937	1,241	1,351	1,166	1,551	106.6	92.2	122.3	98.2	84.6	113.3	-8.0	-13.0	-3.0
Bahia	7,207	6,372	8,159	9,143	7,931	10,424	134.7	119.3	152.9	129.6	112.3	147.7	-4.0	-9.0	2.0
Ceará	3,352	2,929	3,797	4,159	3,588	4,776	104.4	91.0	118.7	95.2	82.1	109.3	-9.0	-14.0	-4.0
Maranhão	2,279	1,977	2,603	2,979	2,574	3,429	106.9	93.0	121.8	101.8	87.9	116.8	-5.0	-10.0	1.0
Paraíba	1,729	1,518	1,964	1,968	1,698	2,258	108.7	94.8	123.8	98.3	84.7	113.1	-10.0	-15.0	-4.0
Pernambuco	3,901	3,429	4,372	4,515	3,937	5,187	123.0	108.5	137.9	107.6	93.8	123.5	-12.0	-17.0	-7.0
Piauí	1,219	1,070	1,392	1,540	1,329	1,795	102.2	89.7	117.0	94.1	81.3	109.7	-8.0	-13.0	-3.0
Rio Grande do Norte	1,225	1,063	1,395	1,548	1,343	1,772	99.5	86.2	113.8	93.9	81.1	107.6	-6.0	-10.0	0.0
Sergipe	658	573	754	866	743	999	98.3	84.9	113.3	92.0	78.8	106.0	-6.0	-11.0	-2.0
**Mid-Western**	4,842	2,416	5,550	5,451	5,770	4,591	102.8	89.1	117.9	93.9	80.5	108.3	-8.8	-13.8	-3.3
Distrito Federal	604	515	697	919	783	1,064	93.3	80.3	107.3	85.6	73.4	98.5	-8.0	-13.0	-3.0
Goiás	2,267	1,970	2,587	3,077	2,644	353	107.8	93.6	123.3	98.4	84.6	113.1	-9.0	-14.0	-3.0
Mato Grosso	998	858	1,144	1,442	1,226	1,671	100.7	87.3	115.5	92.5	78.8	107.2	-8.0	-13.0	-3.0
Mato Grosso do Sul	973	846	1,122	1,300	1,117	1,503	109.4	95.2	125.4	98.9	85.2	114.2	-10.0	-15.0	-4.0
**Southeast**	42,384	38,015	27,400	45,966	41,540	54,216	127.0	113.8	141.7	103.2	90.1	117.6	-18.0	-23.3	-12.8
Espírito Santo	1,378	1,198	1,584	1,780	1,525	2,059	105.2	91.5	119.9	92.7	79.8	106.6	-12.0	-17.0	-6.0
Minas Gerais	9,355	8,344	105	11,325	9,882	12,958	115.1	103.2	129.1	98.6	86.2	112.6	-14.0	-20.0	-9.0
Rio de Janeiro	9,415	8,429	1,047	10,177	8,875	11,582	144.9	131.2	159.9	112.9	98.8	128.3	-22.0	-27.0	-17.0
São Paulo	22,236	20,044	24,664	24,286	21,258	27,617	142.7	129.2	158.0	108.4	95.4	122.7	-24.0	-29.0	-19.0
**Southern**	13,739	12,046	15,521	13,012	10,923	18,534	122.4	107.9	137.8	102.2	88.6	116.7	-16.3	-21.7	-11.3
Paraná	5,074	4,462	5,725	5,923	5,101	6,787	124.4	110.1	140.1	103.0	89.3	117.3	-17.0	-22.0	-12.0
Rio Grande do Sul	6,062	5,317	6,814	6,742	5,819	7,765	128.7	113.6	144.1	103.9	90.2	118.8	-19.0	-25.0	-14.0
Santa Catarina	2,603	2,267	2,982	3,470	3,000	3,982	114.2	100.1	129.3	99.6	86.4	113.9	-13.0	-18.0	-8.0

*Age-standardized rate; U.I.: uncertainty interval.
