# Supplementary material for: Physical inactivity as a risk factor to mortality by ischemic heart disease during economic and political crisis in Brazil
Source: PeerJ. 2020 Oct 15;8:e10192. doi: 10.7717/peerj.10192 (PMC7568855; doi:10.7717/peerj.10192)
Supplement: Supplemental Information 2 — Incidence of ischemic heart disease in Brazilian female population in the years 2007 and 2017 [file peerj-08-10192-s002.pdf]

Supplemental Table S2. Incidence of ischemic heart disease in Brazilian female population in the years 2007 and 2017.

	2007			2017			2007			2017			Change		
	Number	95% U.I.		Number	95% U.I.		Rate*	95% U.I.		Rate*	95% U.I.		% rate*	95% U.I.	
**Brazil**	60,989	53,930	68,671	72,314	62,973	83,031	70.5	62.3	79.4	58.1	50.6	66.7	-18.0	-20.0	-15.0
**Northen**	2,392	2,034	2,764	3,427	2,928	3,983	54.4	46.4	63.0	50.1	42.7	58.5	-7.9	-13.0	-2.7
Acre	104	88	120	152	128	177	56.4	48.2	65.7	51.1	43.5	59.9	-9.0	-15.0	-4.0
Amapá	73	62	85	121	103	142	51.8	43.9	60.5	48.9	41.8	57.7	-6.0	-11.0	-1.0
Amazonas	460	393	531	661	561	772	51.9	44.3	60.1	48.3	41.1	56.3	-7.0	-12.0	-2.0
Pará	1,225	1,045	1,413	1,712	1,470	1,984	55.7	47.6	64.3	51.1	43.5	59.2	-8.0	-13.0	-3.0
Rondônia	238	200	276	354	302	411	55.6	47.8	64.4	50.6	43.3	58.9	-9.0	-14.0	-4.0
Roraima	46	38	54	79	66	93	52.8	45.0	60.7	48.7	41.4	56.6	-8.0	-13.0	-2.0
Tocantins	246	208	285	348	298	404	56.5	48.2	65.6	51.8	44.1	61.0	-8.0	-13.0	-3.0
**Northeastern**	15,630	13,587	17,866	19,356	16,714	22,412	61.8	53.4	71.1	55.7	47.8	64.6	-10.1	-14.7	-4.8
Alagoas	743	639	851	935	802	1,085	60.7	52.0	69.9	54.9	46.9	63.8	-10.0	-14.0	-4.0
Bahia	4,887	4,271	5,538	6,200	5,359	7,188	76.5	66.6	87.0	70.7	61.0	81.9	-8.0	-13.0	-2.0
Ceará	2,381	2,061	2,739	2,953	2,564	3,405	60.3	52.0	69.3	53.8	46.5	62.4	-11.0	-15.0	-5.0
Maranhão	1,344	1,159	1,543	1,777	1,520	2,058	57.2	49.2	65.8	52.8	45.0	61.4	-8.0	-13.0	-3.0
Paraíba	1,262	1,098	1,453	1,446	1,250	1,669	62.7	54.4	72.5	55.3	47.6	63.9	-12.0	-17.0	-6.0
Pernambuco	2,912	2,540	3,327	3,362	2,913	3,892	70.4	61.2	80.5	60.1	51.9	69.5	-15.0	-19.0	-10.0
Piauí	792	686	911	1,011	857	1,173	56.5	48.7	65.3	51.6	43.7	60.1	-9.0	-13.0	-4.0
Rio Grande do Norte	847	737	971	1,072	933	1,247	55.9	48.4	64.4	51.1	44.2	59.7	-8.0	-13.0	-4.0
Sergipe	462	396	533	600	516	695	56.2	47.9	64.8	50.7	43.5	59.1	-10.0	-15.0	-5.0
**Mid-Western**	2,826	2,438	3,267	4,052	3,464	4,705	57.8	49.9	66.7	51.6	44.2	60.2	-10.8	-15.3	-5.5
Distrito Federal	415	354	483	641	544	749	52.5	45.3	60.7	47.0	40.1	54.8	-11.0	-15.0	-6.0
Goiás	1,351	1,171	1,559	1,874	1,604	2,166	62.0	53.9	71.4	54.5	46.8	63.6	-12.0	-16.0	-7.0
Mato Grosso	504	433	584	753	647	880	56.4	48.4	65.1	50.6	43.4	59.0	-10.0	-15.0	-5.0
Mato Grosso do Sul	556	480	641	784	669	910	60.2	52.1	69.4	54.4	46.6	63.3	-10.0	-15.0	-4.0
**Southeast**	30,293	26,892	34,099	34,172	29,666	39,307	70.9	62.8	80.1	57.1	49.3	65.9	-18.8	-24.0	-13.8
Espírito Santo	889	766	1,025	1,161	998	1,356	58.3	50.4	67.1	50.7	43.5	59.0	-13.0	-18.0	-8.0
Minas Gerais	6,559	5,778	7,446	7,766	6,691	8,915	67.4	59.3	76.6	55.9	48.2	64.5	-17.0	-22.0	-12.0
Rio de Janeiro	7,097	6,308	7,986	7,703	6,642	8,859	77.8	69.6	87.2	61.2	52.7	70.4	-21.0	-27.0	-16.0
São Paulo	15,748	14,040	17,642	17,542	15,335	20,177	80.1	71.7	89.5	60.5	52.9	69.8	-24.0	-29.0	-19.0
**Southern**	9,847	8,636	11,214	11,309	9,785	13,097	71.7	63.1	81.5	57.7	50.0	66.8	-19.7	-24.7	-14.3
Paraná	3,342	2,923	3,807	3,962	3,415	4,626	73.3	64.5	83.1	58.2	50.2	67.9	-21.0	-26.0	-15.0
Rio Grande do Sul	4,739	4,178	5,384	5,044	4,374	5,818	76.1	67.3	85.9	58.9	51.2	68.0	-23.0	-28.0	-18.0
Santa Catarina	1,766	1,535	2,023	2,303	1,996	2,653	65.8	57.4	75.6	55.9	48.6	64.5	-15.0	-20.0	-10.0

*Age-standardized rate; U.I.: uncertainty interval.
