# Supplementary material for: Physical inactivity as a risk factor to mortality by ischemic heart disease during economic and political crisis in Brazil
Source: PeerJ. 2020 Oct 15;8:e10192. doi: 10.7717/peerj.10192 (PMC7568855; doi:10.7717/peerj.10192)
Supplement: Supplemental Information 3 — *Age-standardized rate; U.I.: uncertainty interval; SEV: summary exposure value. [file peerj-08-10192-s003.pdf]

Supplemental Table S3. Age-standardized summary exposure value to physical inactivity in the Brazilian male population in 1990 and 2017 in ages ≥ 25 years.

	2007			2017		
	**SEV***	**95% U.I.**		**SEV***	**95% U.I.**	
**Brazil**	57.9	22.9	99.9	58.1	22.9	99.9
**Northen**	56.9	22.6	99.9	57.0	22.6	99.9
Acre	57.4	22.8	99.9	57.6	22.8	99.9
Amapá	58.9	23.1	99.9	58.8	23.0	99.9
Amazonas	56.7	22.6	99.9	56.9	22.6	99.9
Pará	55.4	22.4	99.9	55.9	22.4	99.9
Rondônia	60.0	23.1	99.9	60.1	23.1	99.9
Roraima	55.7	22.4	99.9	55.9	22.4	99.9
Tocantins	53.9	21.9	99.9	54.2	22.0	99.9
**Northeastern**	55.8	22.4	99.9	56.0	22.5	99.9
Alagoas	54.0	21.9	99.9	53.9	22.0	99.9
Bahia	57.0	22.7	99.9	56.9	22.8	99.9
Ceará	57.8	22.8	99.9	57.6	22.8	99.9
Maranhão	51.0	21.1	99.5	51.9	21.4	99.9
Paraíba	57.1	22.7	99.9	57.4	22.7	99.9
Pernambuco	57.1	22.8	99.9	57.5	22.8	99.9
Piauí	55.6	22.4	99.9	55.5	22.4	99.9
Rio Grande do Norte	56.9	22.7	99.9	56.8	22.8	99.9
Sergipe	55.8	22.5	99.9	56.2	22.5	99.9
**Mid-Western**	57.7	22.8	99.9	57.8	22.8	99.9
Distrito Federal	64.7	24.2	99.9	64.8	24.1	99.9
Goiás	55.3	22.4	99.9	55.3	22.4	99.9
Mato Grosso	55.4	22.4	99.9	55.5	22.4	99.9
Mato Grosso do Sul	55.4	22.4	99.9	55.5	22.4	99.9
**Southeast**	59.5	23.2	99.9	59.7	23.2	99.9
Espírito Santo	57.2	22.8	99.9	57.4	22.9	99.9
Minas Gerais	57.2	22.8	99.9	56.8	22.6	99.9
Rio de Janeiro	63.7	24.0	99.9	63.8	24.0	99.9
São Paulo	59.9	23.3	99.9	60.7	23.5	99.9
**Southern**	56.7	22.7	99.9	56.7	22.7	99.9
Paraná	56.8	22.8	99.9	56.6	22.7	99.9
Rio Grande do Sul	55.5	22.4	99.9	55.9	22.5	99.9
Santa Catarina	57.7	22.9	99.9	57.8	22.8	99.9

*Age-standardized rate; U.I.: uncertainty interval; SEV: summary exposure value.
