# Supplementary material for: Physical inactivity as a risk factor to mortality by ischemic heart disease during economic and political crisis in Brazil
Source: PeerJ. 2020 Oct 15;8:e10192. doi: 10.7717/peerj.10192 (PMC7568855; doi:10.7717/peerj.10192)
Supplement: Supplemental Information 4 — *Age-standardized rate; U.I.: uncertainty interval; SEV: summary exposure value. [file peerj-08-10192-s004.pdf]

Supplemental Table S4. Age-standardized summary exposure value to physical inactivity in the Brazilian female population in 1990 and 2017 in ages ≥ 25 years.

	**2007**			**2017**		
	**SEV***	**95% U.I.**		**SEV***	**95% U.I.**	
**Brazil**	59.1	20.9	99.9	59.2	20.9	99.9
**Northen**	59.0	20.9	99.9	59.2	20.9	99.9
Acre	58.6	20.8	99.9	58.7	20.8	99.9
Amapá	61.2	21.3	99.9	61.2	21.3	99.9
Amazonas	59.9	21.0	99.9	60.3	21.1	99.9
Pará	56.1	20.5	99.9	56.5	20.5	99.9
Rondônia	61.2	21.2	99.9	61.2	21.2	99.9
Roraima	59.6	20.9	99.9	59.6	20.9	99.9
Tocantins	56.7	20.6	99.9	57.0	20.7	99.9
**Northeastern**	57.0	20.6	99.7	57.3	20.7	99.9
Alagoas	56.9	20.6	99.9	57.0	20.6	99.9
Bahia	61.0	21.2	99.9	61.0	21.2	99.9
Ceará	58.6	20.8	99.9	58.7	20.9	99.9
Maranhão	48.7	19.1	97.9	49.9	19.5	99.9
Paraíba	57.2	20.7	99.9	57.4	20.7	99.9
Pernambuco	57.4	20.7	99.9	57.7	20.8	99.9
Piauí	58.3	20.8	99.9	58.4	20.8	99.9
Rio Grande do Norte	58.6	20.8	99.9	58.8	20.9	99.9
Sergipe	56.6	20.6	99.9	56.9	20.6	99.9
**Mid-Western**	59.9	21.1	99.9	60.0	21.1	99.9
Distrito Federal	59.0	20.9	99.9	58.9	20.9	99.9
Goiás	61.3	21.2	99.9	61.4	21.4	99.9
Mato Grosso	60.5	21.2	99.9	60.5	21.2	99.9
Mato Grosso do Sul	58.9	20.9	99.9	59.1	21.0	99.9
**Southeast**	59.6	21.1	99.9	59.7	21.1	99.9
Espírito Santo	58.2	20.9	99.9	58.3	20.8	99.9
Minas Gerais	58.5	20.9	99.9	58.5	20.9	99.9
Rio de Janeiro	61.5	21.4	99.9	61.4	21.3	99.9
São Paulo	60.0	21.2	99.9	60.5	21.2	99.9
**Southern**	59.2	21.0	99.9	59.1	20.9	99.9
Paraná	59.6	21.1	99.9	59.2	21.0	99.9
Rio Grande do Sul	58.7	20.9	99.9	58.8	20.9	99.9
Santa Catarina	59.4	21.0	99.9	59.4	20.9	99.9

*Age-standardized rate; U.I.: uncertainty interval; SEV: summary exposure value.
